# Impact of curcumin loading on the physicochemical, mechanical and antimicrobial properties of a methacrylate-based experimental dental resin

**DOI:** 10.1038/s41598-022-21363-5

**Published:** 2022-11-04

**Authors:** Patricia Comeau, Beatriz Panariello, Simone Duarte, Adriana Manso

**Affiliations:** 1 Department of Oral Health Sciences, Faculty of Dentistry, The University of British Columbia, Vancouver, BC V6T 1Z3 Canada; 2 Department of Cariology, Operative Dentistry and Dental Public Health, Indiana University School of Dentistry, Indianapolis, 46202 USA; 3 American Dental Association Science and Research Institute, Applied Research, Chicago, USA

**Keywords:** Dental caries, Biomaterials

## Abstract

Oral biofilms are directly linked to one of the most common chronic human diseases, dental caries. Resin-based dental materials have significant potential to replace amalgam, however they lack sufficient antimicrobial power. This innovative study investigates a curcumin-loaded dental resin which can be utilized in an antimicrobial photodynamic therapy (aPDT) approach. The study evaluated the effects of curcumin loading on resin physicochemical, mechanical, and adhesive properties, as well as the antimicrobial response associated with blue light activation. Preliminary tests involving degree of conversion (DC) and sample integrity determined the optimal loading of curcumin to be restricted to 0.05 and 0.10 wt%. These optimal loadings were tested for flexural strength (FS), water sorption (WS) and solubility (SL), shear bond strength to dentin (SBS), and viability of *Streptococcus mutans* under 14.6 J/cm^2^ blue light or dark conditions, in 6 h and 24 h biofilms*.* The results demonstrated that 0.10 wt% curcumin had minimal impact on either FS or SBS, but detectably increased WS and SL. A 2 log_10_ (CFU/mL) reduction in *S. mutans* after light application in both 6 h and 24 h biofilms were corroborated by CLSM imaging and highlighted the significant potential of this novel aPDT approach with resin-based dental materials.

## Introduction

Oral health plays a significant role in an individuals' general health and well-being, and oral conditions such as dental caries remain a paramount global health concern^1^. Dental caries affects 60–90% of school-aged children and most adults, and remains one of the most prevalent and costly global diseases to date, with the global economic burden of dental diseases totaling $544.41 billion in 2015^2^. Dental caries is the result of a dynamic biological process whereby microorganisms, microbial products, host (saliva), and diet (sugar) interact on the tooth surface and lead to the establishment of pathogenic biofilms (dental plaque) with eventual dental carious lesions^3^. The loss of tooth structure allows niches to form where the biofilm is not easily accessible and further challenges effective, long-lasting caries management. Minimally invasive restorative procedures aim to remove this faulty and highly infected dental tissues and replace this lost tissue with materials presenting suitable mechanical and biological properties for clinical service.

Dental amalgam, while being an effective restorative dental material for more than one hundred years, has been recommended for a “phase down” during the Minamata Convention (United Nations, WHO) in 2013^4^ due to the potential for human health risk and environmental damage associated with improper use and waste management. The Convention also made recommendations, including the exploration by the scientific communities of alternative materials that could effectively replace the dental amalgam. As such, resin-based dental materials have been of significant research interests in an effort to effectively identify alternative materials with acceptable, long-lasting mechanical and antimicrobial properties. In parallel, it is important to highlight that there is an increased risk of secondary caries associated with resinous materials due to a combination of factors^5^. For instance, dental resins are considered biofilm-prone surfaces due to their physicochemical characteristics^6,7^ and to date have often shown poor antimicrobial and antifouling properties^8,9^. In fact, certain types of unreacted monomers and degradation by-products present in resins may alter virulence factors and promote the growth of several bacterial strains^10,11^. In addition, the presence of matrix metalloproteinases (MMPs)^12^ and the inherent polymerization shrinkage^13^ at the resin-dentin interface may lead to the formation of a gap more susceptible to the accumulation of cariogenic biofilms and development of secondary caries.

A common microorganism related to dental caries is *Streptococcus mutans* (*S. mutans*), which is responsible for the early attachment of the cariogenic biofilm and plaque formation upon frequent exposure to dietary sucrose^14,15^. Current plaque-related disease management involves mechanical scouring of the pathogenic biofilm and the use of antibacterial agents if needed^16^. However, the universal application of potent antimicrobial agents such as chlorhexidine rinse is limited by its side effects including bitter taste^17,18^, teeth staining^19^, allergic reactions^20,21^, and acquisition by oral bacteria of antimicrobial resistance^22,23^. In addition, a complete and efficient mechanical biofilm disruption is not always possible. Therefore, there is a significant need to explore new approaches and resin-based restorative materials are promising candidates, which can both prevent secondary carious lesions and assist in the management of the pre-existing disease more effectively. Several studies have previously shown that cariogenic bacteria such as *S. mutans* are susceptible to antimicrobial photodynamic therapy (aPDT)^15,24,25^ involving three key elements: a photosensitizing agent (PS), oxygen, and light. In this approach the selected PS is activated by the applied light with a matching wavelength, and singlet oxygen and/or other reactive oxygen species (ROS) are produced^16^. These ROS then act on the bacterial cell membrane, wall, or DNA to produce oxidative damage^16^. Each of the three aPDT components is generally harmless by itself but upon combination can produce an antimicrobial effect. So far, very few studies have incorporated photosensitizers into dental methacrylates aiming to achieve a target-specific, repeatable, and sustainable aPDT^26^. This study goal is to further explore the aPDT approach associated with dental resins for restorative applications.

Prior research has shown that light properties such as wavelength, output power, and beam diameter significantly impact the efficacy of aPDT^24^. Nowadays, light-emitting diodes (LEDs) are the most common light source owing to their compact size and portability, low operation cost, and ease of use. In addition, LED sources in the blue wavelength are readily available in routine dental practice. Thus, this study used a blue LED light-curing unit (LCU), a safe and commonly utilized light source for photopolymerization of methacrylate-based materials. The natural PS selected for this study, curcumin, was then chosen based on its ability to match the specific light spectrum of the selected LCU (380–520 nm)^24^ and theoretically generate adequate light potency^27^.

Several PS’ have been shown to be active within the blue light spectrum, including rose Bengal (RB), eosin (EOS), erythrosine (ERI), and curcumin. Of these, curcumin is a very promising natural PS for aPDT with many previously proven pharmacological effects, including anti-carcinogenic, antioxidant, anti-infection, and anti-inflammatory properties^25^. It has a molecular weight of 368.38 g/mol, has some of the highest light absorption of available PS’ in the blue wavelength range (300–500 nm; one maximum at ~ 450 nm)^27^, and while relatively insoluble in water, may be dissolved in organic solvents such as *n*-methyl-glucamine, ethanol, and DMSO solutions^28,29^. It has also previously been shown to have an antimicrobial activity following aPDT against both cariogenic planktonic cells^25,30,31^ and, to a lesser degree, cariogenic biofilms^28,30,32,33^ when applied as a solution. Furthermore, curcumin has been shown to either have no inhibitory effect^34^ or promote cell viability^35^ of dental pulp cells. While these earlier investigations have shown the significant potential of curcumin as a PS in aPDT in direct contact with the cells, the prevention and management of dental caries would significantly benefit from a more site-specific and sustained antimicrobial effect, particularly on critical tooth-restoration interfaces susceptible to recurrent caries.

Loading particles and compounds in novel dental resinous materials must undergo a series of screening laboratory testing before they may be deemed suitable to clinical trials and further address specific clinical needs. The degree of conversion (DC), which is related to changes relative to the initial reactive group (e.g. methacrylate) concentration, is an example and represents a significant early determinant of resin properties and performance in situ^36,37^. In addition, upon loading with particles and/or compounds, these materials must sustain or improve certain properties associated with its clinical uses, such as flexural strength (FS), shear bond strength (SBS), and water sorption/solubility (WS/SL) to survive the challenges posed by the oral environment for many years. Finally, to address the purposes of an antimicrobial material, it must also have sufficient antimicrobial properties. Thus, to address the clinical needs associated with dental caries, the curcumin-loaded resin system was tested for DC, FS, SBS, WS/SL, and antimicrobial response of *S. mutans* biofilm. It is hypothesized that there is a maximum curcumin concentration beyond which the degree of conversion is negatively impacted, while within these bounds, the mechanical and adhesive properties are not affected. Concurrently, it is hypothesized that the inclusion of curcumin in the resin formulation will improve the antibacterial properties following application of blue light in an aPDT strategy.

## Results

### Initial screening of different curcumin concentrations added to resin blend

**Figure 1:**
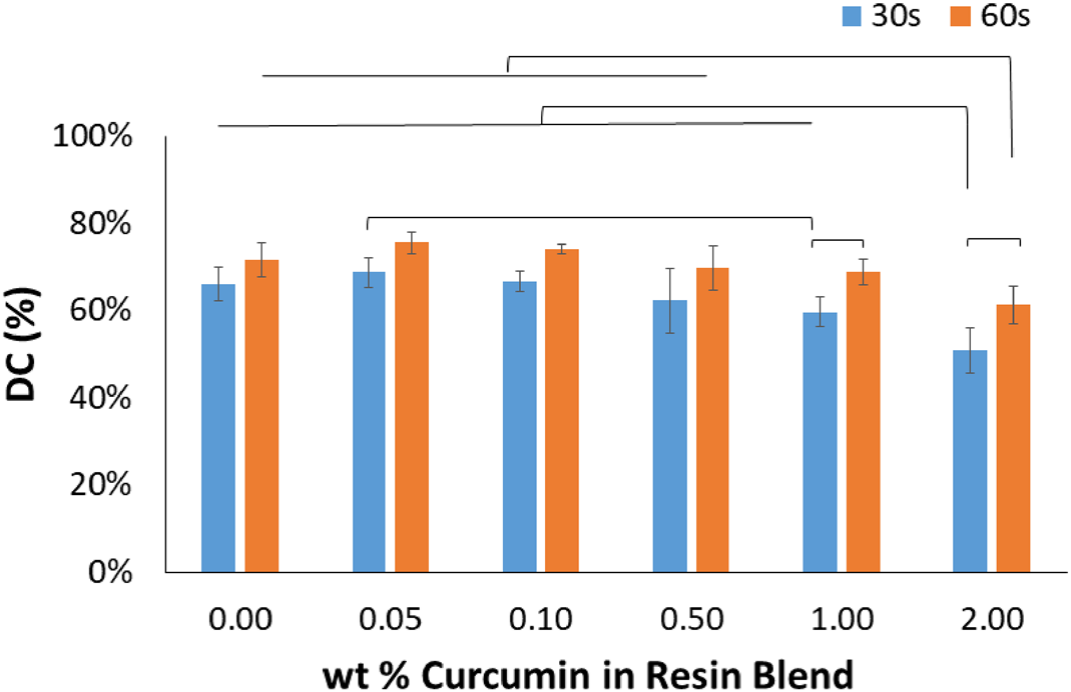
Degree of conversion of resin blends as a function of curcumin concentration and light curing time. Data is presented as average (%) ± one standard deviation (n = 6). Horizontal bars indicate a statistically detectable difference (p < 0.05).

FTIR analysis confirmed that both curcumin concentration and light curing time impacted the degree of conversion (DC) of the experimental resin blends (p < 0.001 for both; Fig. 1).

**Figure 2:**
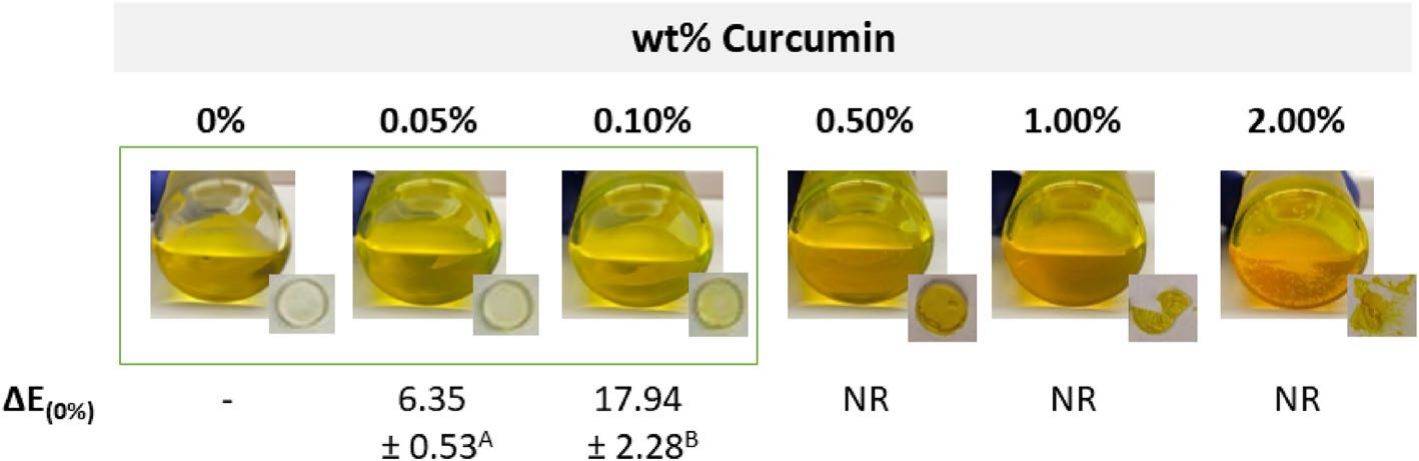
Images of the pre-polymerized blends with inset images of the experimental resin-blend disks after 60 s light curing on each side. The disks were classified as completely polymerized for the 0%, 0.05% and 0.10%, inconsistent for the 0.5%, or incomplete for 1% and 2%. The difference in colour (∆E_(0%)_) is reported for the 0, 0.05 and 0.10 wt% curcumin-loaded disks relative to the unloaded (0% curcumin) disks; the colour of disks of higher concentration was not recorded (NR). Means that are detectably different (p < 0.05) are assigned different letters.

The addition of 2.00 wt% curcumin to the resin blend resulted in a detectable drop in DC compared to the unloaded blend (p < 0.001 for 30 s, and p = 0.003 for 60 s). Meanwhile, in a concurrent check of disk stability following standard fabrication protocol with 60 s light cure on top and bottom surfaces, it was found that more than a 0.50 wt% of curcumin added to the blend resulted in inconsistent and unstable disks (Fig. 2). As a result of both DC and analysis of the disks’ physical integrity, further study was limited to 0, 0.05, and 0.10 wt% curcumin added to the resin blend.

The addition of an increasing amount of curcumin to the blend notably increased the yellow-orange pigmentation of the samples, with the difference in colour from the 0% disks (∆E_(0%)_) dependent on the curcumin concentration (p < 0.001).

### Shear bond strength to dentin (SBS)

**Figure 3:**
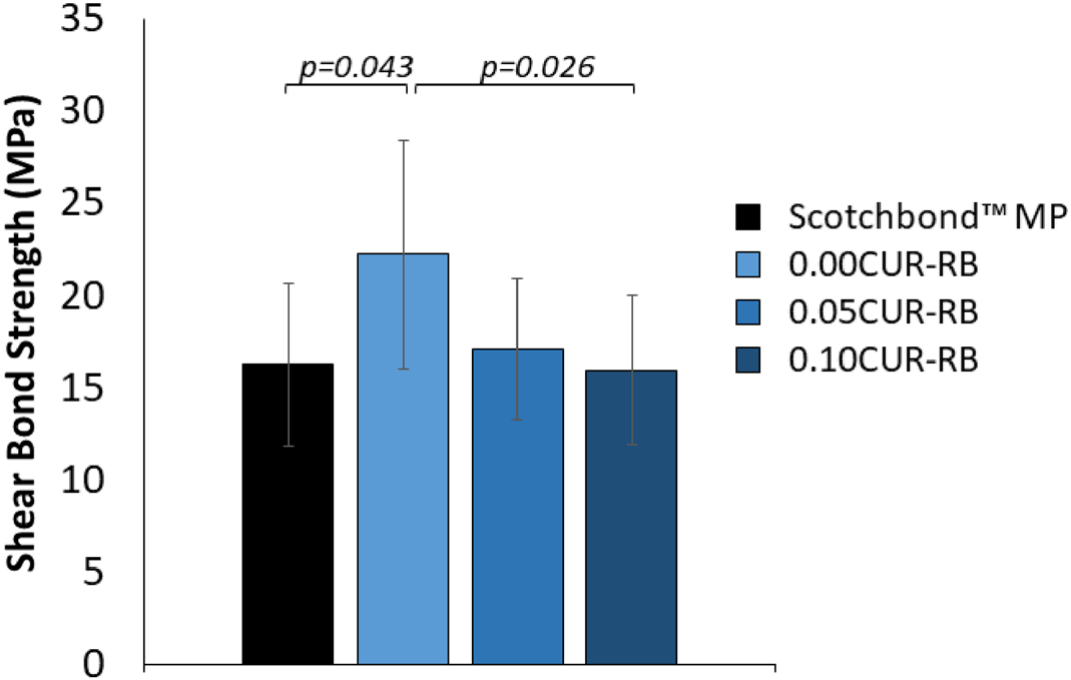
Shear bond strength to dentin as a function of curcumin concentration. Scotchbond MP served as a study commercial control. Data reported as average (MPa) ± one standard deviation (n = 12). Horizontal bars indicate statistically detectable differences (p < 0.05).

The shear bond strength (SBS) of the experimental resin blends was detectably impacted by the inclusion of curcumin (p = 0.017, Fig. 3).

While there was a detectable drop in SBS upon adding 0.10 wt% curcumin to the resin blend (p = 0.026), only the unloaded resin blend (0%) had a detected change in SBS compared to the commercial Scotchbond MP study control. Overall, the impact of curcumin on SBS of the resin blend was minimal and remained comparable to a well-known commercial product.

### Flexural strength (FS)

**Figure 4:**
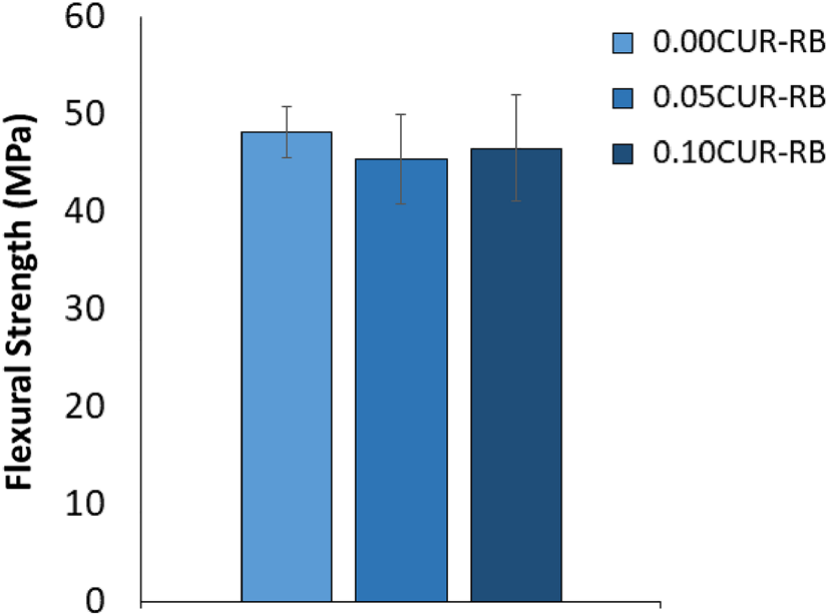
Flexural strength as a function of curcumin concentration. Data reported as average (MPa) ± one standard deviation (n = 13). No statistically significant differences were detected.

There was no detected dependence of flexural strength (FS) on curcumin inclusion (p = 0.278, Fig. 4).

### Water sorption and solubility (WS and SL)

**Figure 5:**
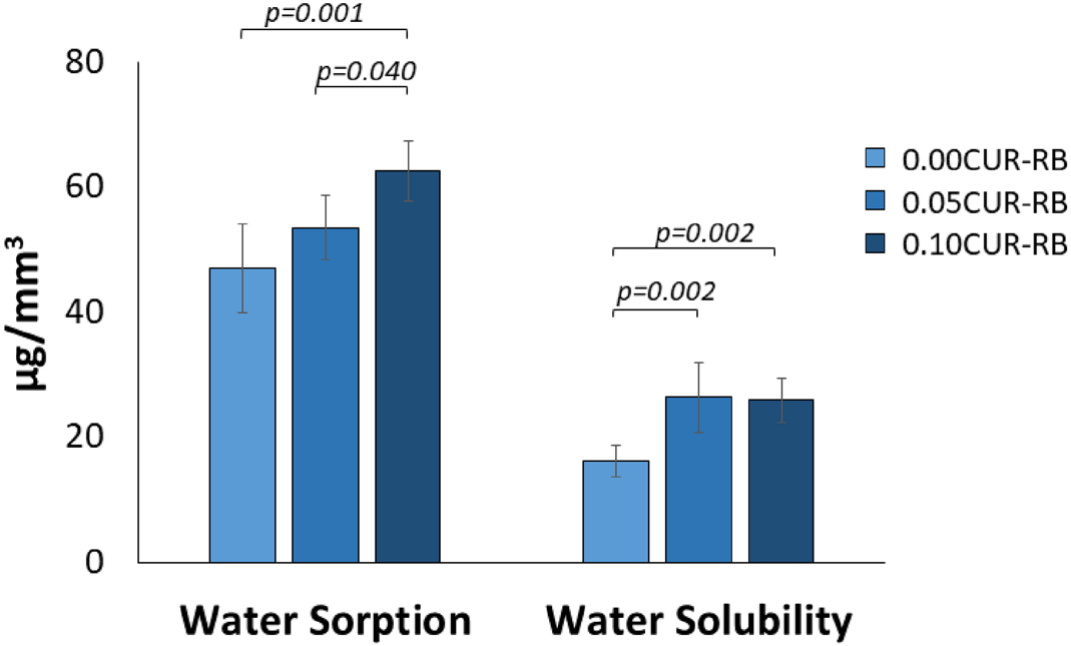
(Left) Water sorption and (right) Water solubility of the experimental resin blends as a function of curcumin concentration after 14d in 37 °C water. Data is presented as average (µg/mm^3^) ± one standard deviation (n = 6). Horizontal bars indicate statistically detectable difference (p < 0.05).

The water sorption and solubility of the resin disks was significantly impacted by the curcumin concentration (p = 0.001 and p < 0.001, respectively; Fig. 5).

The addition of 0.10 wt% curcumin to the experimental resin blends significantly increased the water sorption compared to both the 0 wt% and 0.05 wt% blends (p = 0.001 and 0.040, respectively). Meanwhile, the resin blends containing 0.05 wt% and 0.10 wt% showed greater solubility than the unloaded disks (p = 0.002 for both).

### *S. mutans* CFU/mL count response assay

**Figure 6:**
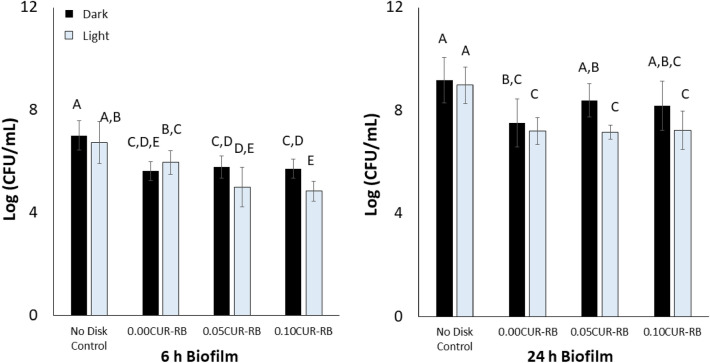
CFU/mL count as a function of curcumin concentration and application of light following 6 h (left) and 24 h (right) biofilm development. Data is presented as average (CFU/mL) ± one standard deviation (n = 9). Means that are detectably different (p < 0.05) are assigned different letters.

The 2-factor general linear model analysis showed that the addition of curcumin and the application of 14.6 J/cm^2^ blue light each independently impacted the CFU/mL count at 6 h and 24 h (p < 0.001 for all; Fig. 6). More specifically, after 14.6 J/cm^2^ of light both curcumin-loaded RBs had detectably lower 6 h CFU/mL count than the unloaded specimens (p = 0.012 for 0.05% and p = 0.002 for 0.10%). Meanwhile, 6 h biofilm on 0.10% and 24 h biofilm on 0.05% had detectably lower CFU/mL after 14.6 J/cm^2^ of light than the dark specimens (p = 0.028 and p = 0.014, respectively).

### Confocal live/dead assays

**Figure 7:**
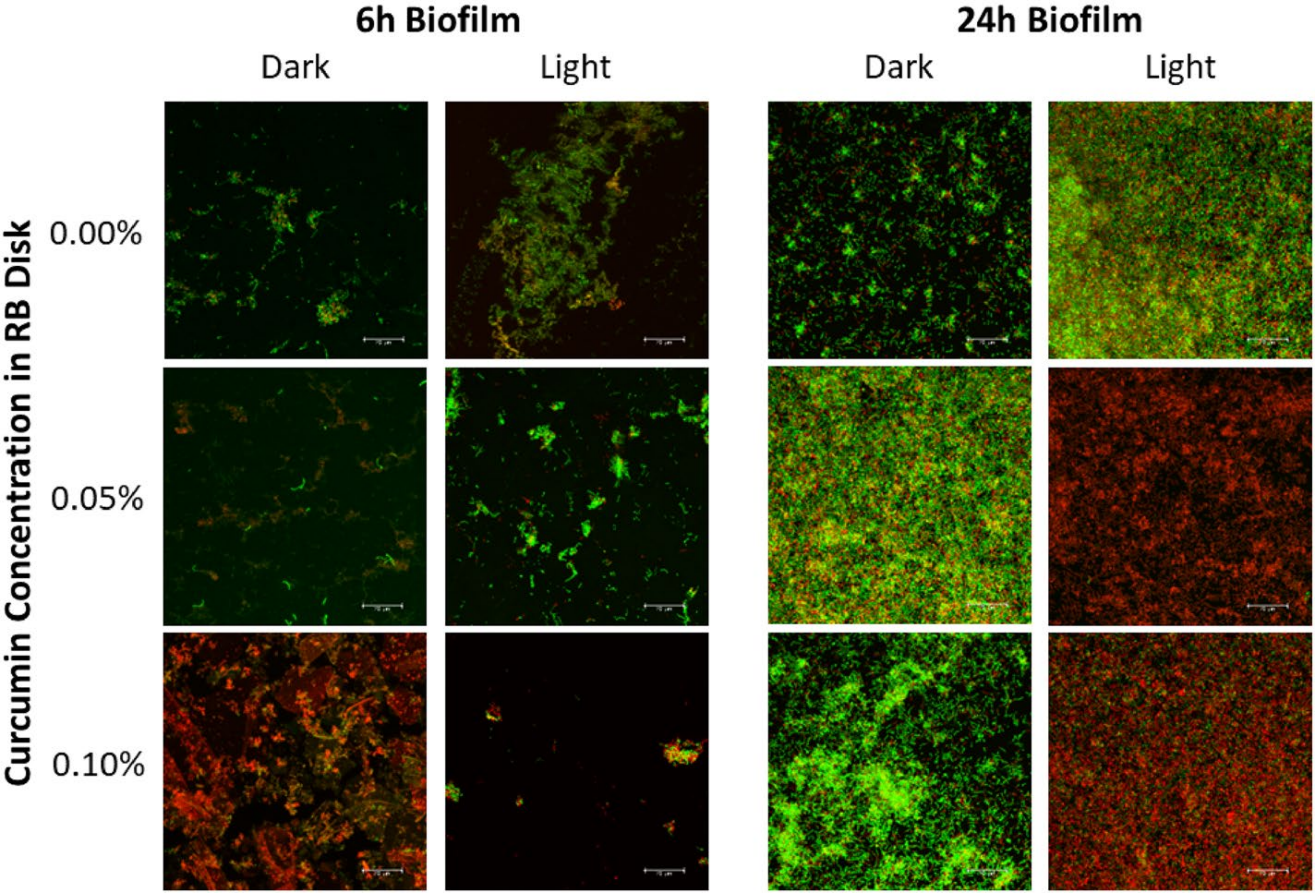
CLSM images of resin disks as a function of curcumin concentration and application of light.

The CLSM images (Fig. 7) confirmed the CFU results (Fig. 6), where it was visually observed that the presence of CUR in the disks are represented by more red (dead) than green (live) cells. Moreover, 6 h biofilms visually displayed less bacteria compared to 24 h biofilms, and the presence of light visually resulted in more red (dead) cells than green (live) cells.

### Curcumin release from resin disks

No curcumin was detected in the eluent following 6 h of the disks being stored in 37 °C water, however 2.56 ± 1.21 ppm was detected in the eluent after 24 h under the same conditions. These results indicate that the disks are very stable in an aqueous environment up to at least 24 h (as designed to match the bacteria and biofilm investigation) and the bacteria and biofilm response to curcumin is likely due to its presence at the sample surface.

## Discussion

While resin-based dental materials are promising restorative alternatives to dental amalgam, they lack antimicrobial power to contribute to disease management themselves. To address this clinical need, we propose the development of an innovative photosensitizer-loaded antimicrobial resin-based dental material for restorative purposes, which can be associated with aPDT to target cariogenic bacteria such as *S. mutans*.

In dentistry, most resin-based restorative products utilize the same methacrylate-based family of monomers and photo-polymerization-based mechanism^38^. The optimal polymerization of such resins is crucial for the final material’s properties and, by consequence, its clinical performance. The DC is a very useful and powerful characterization method as it directly measures the conversion of monomers into polymers, and has been found to directly correlate to the observed physical, chemical and mechanical properties of resins^36,37^. In this study, the DC of all formulations were kept well above 55%. However, a curcumin loading of 2.0 wt% resulted in a significant decrease in DC by 10–15%. It is possible that the higher concentrations of curcumin competed for the blue light initially applied for the photopolymerization as both curcumin and camphorquinone absorb similar wavelength light^27,39,40^. In addition, successful light curing of the 1 mm-thick disk specimens was limited even further to a loading of less than 0.5 wt% curcumin. From this observation, it is also plausible that the presence of undissolved curcumin may have affected the light transmission through the thickness of the specimen and further limited the action of the photoinitiator due to light scattering and attenuation^41,42^.

As the anticipated application and main benefit of this adhesive resin blend will be at dental-composite interfaces for direct and indirect restorations in posterior teeth (the most prone interfaces for secondary caries), the aesthetics and appearance of the material is not considered critical given the proposed benefits. However, as a further useful characterization, the colour differences as a function of curcumin concentration were also characterized. Here, as the curcumin concentration in the blend increased, the colour difference from the unloaded, 0% blend also increased.

A photoinitiator system is essential to obtain a final resin-based dental material suitable for clinical applications in Dentistry. The d,l-camphorquinone (CQ)/tertiary amine system is the most commonly used option for materials available in the market, and it was the system used in this study. While CQ is the chemical responsible for triggering the polymerization reaction, its rate is very low and amines are added as co-initiators to accelerate the process^43^. The CQ actively absorbs the blue applied light as a sensitizer, but in the presence of a tertiary amine (the hydrogen-donating agent) a photoexcitation complex is formed, which subsequently generates amine-derived free radicals^44^. Curcumin may produce weak acids during degradation^29,45^ that tertiary amine groups could subsequently interact with. Thus, it is theoretically possible that the curcumin present in the experimental resin blends could cause a time delay after the light activation of the resin and result in a decrease in DC. In this study, FTIR spectra did not show any significant changes beyond that expected upon light application (i.e., drop in aromatic carbon) and new small peaks attributed to curcumin itself. Future investigation will need to consider DC (and FTIR spectra) response to altering both curcumin and photoinitiator loading concentrations, as well as the use of other characterization (such as UV/Vis)^46^ to observe any impact on resin light absorbance. Interestingly, during fabrication it was observed that loading 0.1% or more resulted in the presence of ever-more undissolved curcumin powder in the resin formulation. As a result, within the boundaries of this study, the presence of undissolved powder likely had a greater impact on DC than any side reaction of curcumin, which can be potentially related to the light scattering associated with the undissolved compound. Other studies have similarly found a decrease in DC with increased PS, and these authors commonly attribute this decrease to the formation of undissolved PS agglomerates^47,48^.

The curcumin-loaded resin blend must also provide similar or superior mechanical, physical and adhesive properties, when compared to the unloaded blend. In this study, the impact of curcumin loading (up to 0.1 wt%) on flexural strength (FS) and shear bond strength (SBS) was minimal after immersing the prepared samples in 37 °C water for 24 h. Additionally, the experimental blends presented with an equivalent SBS to dentin when compared to a commercial product, SBMP, used as a control for the shear bond strength tests. Unfortunately, there are few comparable studies which have loaded other soluble photosensitizers into acrylate- or methacrylate-based materials, but those that have also typically not found any significant change in mechanical or physical properties^49^. However, when near the threshold of solubility, any undissolved PS may act more as a filler and the resultant interfacial interaction between the undissolved particles and the resin matrix must then also be considered. A weaker interface between particles and resin matrix has previously been found to decrease any gains in particle loading on mechanical and physical properties^50^. To a small degree, this was observed in the SBS results in this study, with 0.1%-loaded RB having a detectably lower SBS than the unloaded blend.

While the ideal dental polymer should be relatively insoluble with high chemical and thermal stability, in practice most materials do absorb some water and chemicals as well as release them to the surrounding oral environment^51^. The chemistry and chain length of a polymer are significant determinants of its hydrophilic/hydrophobic nature. The presence of certain functional groups in a polymeric material, such as hydroxyl, carboxyl, and phosphate, tend to increase its hydrophilicity^52^. The resin blend used in this study consists of a mix of different monomers, with varying degrees of hydrophilicity, which led to water sorption and solubility values much lower than that found for commercial adhesives over a longer study time^51^. It is only upon adding curcumin that both water sorption and solubility notably increase, with the addition of 0.1% curcumin resulting in an almost 50% increase in both properties compared to the unloaded resin blend. As noted with the analysis of SBS, the presence of a few undissolved curcumin particles in the 0.1% curcumin resin blend specimens likely contributed to the increased water sorption and solubility due to the creation of a weak interface between undissolved PS particles and resin matrix. This would in turn allow unbound water molecules to readily diffuse in and out of any resultant voids within the polymer^53,54^, as well as increase the ability of water to interact with any polar groups within the resin specimens. It is important to also note that the mass recorded in the solubility measurements reflects the loss of any chemicals (e.g., unreacted monomers, photoinitiators, and undissolved compound) from the polymerized resin specimens. As a result, with enhanced water influx and efflux, there was likely a greater efflux of such chemical species from the curcumin-loaded specimens than the unloaded during the 14-day analysis.

*Streptococcus mutans* have been extensively studied as a screening microorganism during the development of dental materials with antimicrobial properties against cariogenic biofilms. *S. mutans* are directly involved in the early attachment and pathogenesis of cariogenic biofilms^55,56^. In this study a single species microbiological evaluation model using *S. mutans* was evaluated following 6 h and 24 h of biofilm growth in response to different curcumin concentration within the resin specimen (0, 0.05, and 0.10 wt% curcumin) and different blue light conditions (0 or 14.6 J/cm^2^). The dark toxicity (i.e. no light) was minimal, with curcumin-loaded samples resulting in a notably lower log_10_ reduction in *S. mutans* than the unloaded (0% curcumin) samples when compared to the respective test control (no resin disk). It is likely that eluents from the fresh resin disks may have an impact on the behaviour of the selected microorganism^57^, such as its early attachment, growing and/or killing conditions. However, this evaluation was not part of this study and should be considered for future investigation in order to confirm what chemical species (e.g. unreacted monomers, photoiniators) may be eluting from the freshly made resin specimens. In addition, it is possible that steam sterilizing the samples may have induced a change to the samples^58^ and as such, future investigation should also consider the significance of different sterilization techniques. Here, all samples underwent the same sterilization protocol and such potential impact may then be similar for all experimental groups. Meanwhile, following 14.6 J/cm^2^ of blue light application, all curcumin-loaded specimens exhibited an approximate 2 log_10_ (CFU/mL) reduction after 6 and 24 h biofilm growth. While this is not the 3 log_10_ reduction considered biologically relevant by the American Society for Microbiology and CLSI standard^59^, it is comparable to what has previously been reported for pre-established biofilm models^28,32^. In such models, curcumin is provided in solution form and the blue light is applied, albeit at various curcumin concentrations and blue light energies. Planktonic suspensions have responded with anywhere from 2 to 5 log_10_ reduction following application of a curcumin solution and blue light^25^. While it is known that aPDT effectiveness is minimized by the presence of biofilm because of various virulence factors^30^, such evidence serves as the motivation to seek further improvement in PS-loaded material design and selection of optimal blue light application. In fact, recent studies have indicated that the curcumin binds preferentially to the lipid membrane of the bacteria^60,61^. Thus, the proposed aPDT approach is challenged by the ability of the selected PS to interact with the bacterial membrane, to penetrate and act inside the cells, and to produce ROS following illumination. Also, the likelihood that sufficient curcumin will reach the bacteria by eluting from the restorative into the oral environment is remote. As shown in this study, very small amounts of curcumin were released from the disks (only ~ 2 ppm after 24 h). As such, most of the antimicrobial effects observed may be attributed to the presence of curcumin on the material’s surface. Future studies will need to further evaluate the impact of resin blends chemistry and design to increase curcumin loading for a stronger antibacterial response, as well as use chromatography to evaluate the release of any organic compounds (in addition to curcumin) from the system^62^. Also, modifications in the blue light parameters with increased exposure time and/or energy delivered that could potentially enhance the aPDT effects needs to be further evaluated. Furthermore, future investigations will also assess the antimicrobial responses on more mature biofilms (single and dual species) to curcumin-loaded materials, conduct cytotoxicity assays with fibroblasts and odontoblastic cells, and pursue additional in situ investigations. While the aim of the proposed approach is not to permanently eradicate all bacteria, proper caries management entails reaching and subsequently maintaining a healthy oral microbiological consortium within the mouth.

## Conclusion

The feasibility of the proposed concept of using a curcumin-loaded methacrylate-based resin system for aPDT was demonstrated in this study. The maximum optimal loading of curcumin based on DC and 1-mm thick sample fabrication was found to be 0.10 wt%. The selected curcumin loading in the resin had a minimal impact on flexural strength or shear bond strength results, and a 2 log_10_ (CFU/mL) reduction in *S. mutans* in either 6 h or 24 h biofilm following aPDT. As both water sorption and solubility increased with greater curcumin loading, future investigations will seek to tune the resin chemistry with the aim of reducing its hydrophilicity; this would in turn make the resin specimens more stable in aqueous environments. Concurrently, future studies will work on optimizing the resin chemistry and blue light parameters towards increasing the antibacterial response further. This is the first study detailing the loading of curcumin in a methacrylate-based resin system and indicates the significant potential for the proposed approach to address caries management.

## Materials and methods

### Preparing curcumin-loaded resin blends

An experimental dental resin-blend (RB) was prepared using tetraethylene-glycol-dimethacrylate (TEEGDMA, 30 wt%), ethoxylated (4) bisphenol A Dimethacrylate (Bis-EMA, 50 wt%), 2-hydroxypropyl-methacrylate (HPMA, 14 wt%), ethanol (EtOH, 4 wt%), and a photoinitiator system consisting of camphorquinone (CQ, 0.66 wt%) and ethyl 4-(dimethylamino) benzoate (Amino, 1.34 wt%). The RB was loaded with curcumin (Santa Cruz Biotechnology, Texas, USA) at 0.05, 0.1, 0.5, 1.0, and 2.0 wt%, and the unloaded RB (0% curcumin) served as a study control for all tests. Curcumin was added at the requisite amount to the 0% resin blend in a glass vial, and stirred on a magnetic stir plate for approximately 1 h in the dark at room temperature. Immediately prior to sample preparation the vial was vortexed at high speed for a minimum of 30 s. TEEGDMA, Bis-EMA, and HPMA were purchased from Scientific Polymer Products Inc. (Ontario, New York, USA), while CQ and Amino were purchased from Sigma Aldrich, Inc (St. Louis, Missouri, USA).

### Initial screening of different curcumin concentrations added to resin blend

All five experimental blends and control (n = 6) were analyzed by FTIR (IRPrestige-21, Shimadzu, Kyoto, Japan) for degree of conversion (DC). Here, a drop of each formulation was placed between two pieces of polyacetate film, the film was placed in a direct-light pass-through sample holder, and 0, 30 or 60 s of light applied with a Bluephase light curing unit (LCU; Ivoclar Vivadent, Schaan, Liechtenstein) at ~ 1300 mW/cm^2^. The tip of the LCU was positioned directly at the film surface. Spectra was collected over a range of 400–4000 cm^−1^ at a resolution of 2.0 cm^−1^ and with 64 scans under transmittance mode. Sample thickness was ~ 0.05 mm for this characterization only. The DC was calculated in accordance with Eq. (1).

1 $$\mathrm{\% DC}\hspace{0.17em}=\hspace{0.17em}\left(1- \frac{{\frac{absorbance\left(1636cm-1\right)}{absorbance\left(1608cm-1\right)}}_{light-cured}}{{\frac{absorbance\left(1636cm-1\right)}{absorbance\left(1608cm-1\right)}}_{uncured}}\right)\times 100\mathrm{\%}$$

Using PVS molds (6 mm-diameter × 1 mm-thickness), disks were fabricated with each blend and photopolymerized for 60 s on each side with a Valo LCU (Ultradent Products, Inc., UT, USA) at 830 mW/cm^2^. Altogether, the DC analysis and the assessment of the physical integrity of the disks served as an early screening of curcumin-added experimental RB, and two optimal formulations (0, 0.05, or 0.10 wt% curcumin) were further characterized against the unloaded control blend (0%).

### Quantifying colour difference using CIELab colour system

6 mm-diameter × 1 mm-thick disks of the optimal blends (0.05 and 0.10 wt% curcumin) and the control blend were fabricated as described previously (n = 6). All disks were measured 5 times with a VITA Easyshade V (Vita Zahnfabrik H. Rauter GmbH & Co., Germany), with the coordinate values for L*, a*, and b* recorded. L is the lightness variable, while a* indicates where the colour sits on the red/purple-green/blue axis and b* the position along the blue/purple-yellow axis^63^. The difference between the two blend colours (i.e. ∆E_ab_) was further quantified by calculating the differences in L* (i.e. ∆L), a* (i.e. ∆a), and b* (i.e. ∆b) between each optimal blend and the control (0%) blend and entering these values in to Eq. (2).

2 $${\Delta \mathrm{E}}_{ab}= \sqrt{{\Delta \mathrm{L}}^{2}+{\Delta \mathrm{a}}^{2}+{\Delta \mathrm{b}}^{2}},$$

### Shear bond strength to dentin (SBS)

Extracted sound human molars were first sectioned coronally and polished with 600-grit SiC sandpaper to expose mid-coronal dentin. Each surface was then etched (15 s phosphoric acid), rinsed, primed (15 s active application of 3 M ESPE Adper Scotchbond multi-purpose primer), bonded with one experimental RB (0, 0.05, or 0.10 wt% curcumin) or the commercial control (3 M ESPE Adper Scotchbond MP multi-purpose adhesive), and light-cured (20 s) with the Valo LCU at 830 mW/cm^2^. An Ultradent jig was used to create a composite build-up (Estekute Ƹquick Tokuyama Dental Corp., Japan) and light-cured for 40 s. After 24 h in 37 °C MQ ultrapure-water, specimens were tested in shear mode (BISCO tester, Bisco Canada, Richmond, BC, Canada) at 0.5 mm/min (n = 12). The SBS experiment was performed in accordance with relevant guidelines and regulations, and approved by the University of British Columbia’s Human Research Ethics Board before conduction (REB-H14-02189). The informed consent for the extractions was obtained by the clinicians performing the procedure. No additional informed consent was obtained for this protocol, as approved by the Human Research Ethics Board at UBC, as the clinical reasons for extraction have no bearing on the research protocol performed.

### Flexural strength analysis (FS)

Flexural strength (FS) bars (25 mm long × 2 mm × 2 mm) were fabricated for each experimental RB (0, 0.05, or 0.10 wt% curcumin) in Teflon molds and light-cured for 120 s total (60 s at both top and bottom surfaces) with a Valo LCU at 830 mW/cm^2^. The FS bars were then stored in 37 °C MQ ultrapure water for 24 h and tested on a Universal Testing Machine (Shimadzu, Kyoto, Japan) at 1 mm/min in accordance with the ISO 10477 standard (n = 13).

### Water sorption and solubility analysis (WS and SL)

Resin disks (6 mm-diameter × 1 mm-thickness) were fabricated using PVS molds and light cured as described previously (n = 6). The mass (m_1_) and volume (V) of the resin disks (0, 0.05, or 0.10 wt% curcumin) were then recorded before adding them to 37 °C MQ ultrapure water for 14 days. At regular time points, each disk was dried, weighed, and returned to the 37 °C ultrapure water. After 14 days, the disks were weighed (m_2_) and then stored for drying in a sealed container containing desiccant within a 37 °C incubator until a constant weight was reached (m_3_) (i.e. < 1 mg change in mass in three consecutive readings). The WS and SL were then calculated in accordance with Eqs. (3) and (4), respectively^51^.

3 $$\mathrm{WS}\hspace{0.17em}=\hspace{0.17em}\frac{{m}_{2}- {m}_{3}}{V},$$

4 $$\mathrm{SL}\hspace{0.17em}=\hspace{0.17em}\frac{{m}_{1}- {m}_{3}}{V}.$$

### Bacterial strain and growth conditions

A standard strain of *Streptococcus mutans,* UA159 from the American Type Culture Collection (ATCC 700610; Rockville, MD, USA), was used to form the single-species biofilms. Stock cultures were maintained at − 80 °C, reactivated onto 5% Sheep Blood Agar plates (BBL, Becton, Dickinson and Company, Sparks, MD, USA) and incubated at 37 °C for 48 h. After that, for the formation of the pre-inoculum, the bacteria were individually reactivated by transferring single colonies (10–12) to a tube containing 5 mL of brain–heart infusion (BHI) broth culture medium (BD BBL, Becton, Dickinson and Company, Sparks, MD, USA) supplemented with 1% glucose and kept overnight in an incubator (5% CO_2_, 37 °C). Inoculum started from an absorbance of 0.08–0.10 read at an optical density (OD) of 600 nm (BioTek, Winnoski, Vermont, USA), corresponding to 1.5 × 10^8^ colony forming units (CFU)/mL. The inoculum was diluted with BHI broth to obtain a final *S. mutans* concentration of 1 × 10^6^ CFU/mL.

### *S. mutans* biofilm formation and treatments

Additional resin blend (0, 0.05, 0.10 wt% curcumin) disks (6 mm-diameter × 1 mm-thickness) were prepared for evaluations of the antimicrobial activity as previously reported using PVS molds and the Valo LCU (830 mW/cm^2^). The disks were then autoclaved at 121 °C for 30 min and then incubated with 0.5 mL of the diluted *S. mutans* inoculum at 1 × 10^6^ CFU/mL in 24-well plates (Thermo Fisher Scientific, Rochester, NY, USA) at 37 °C (5% CO_2_) (Isotemp CO_2_ incubator, Thermo Fisher Scientific, Marietta, OH, USA). The biofilms were kept undisturbed for the allocated time, 6 h or 24 h, to allow biofilm formation at 37 °C under microaerophilic conditions. After each tested biofilm period (6 or 24 h), the samples were washed with 1 mL of sterile phosphate buffered saline (PBS) and the following treatments were performed: blue light-treatment (“Light”) for 60 s at a distance of 18 mm from the sample, which is equivalent to an energy density of 14.6 J/cm^2^ (Valo at 830 mW/cm^2^; λ = 385–515 nm) and no light-treatment (“Dark”).

### *S. mutans* CFU count response assay

After treatments, the resin disks were transferred to pre-labelled microcentrifuge tubes containing 1 mL sterile phosphate buffered saline (PBS) and sonicated for 30 s at amplitude 20 (QSonica sonicators, Newton, CT, USA). Serial dilutions, by factors of 10 until a final serial dilution of 10^–7^, were performed in sterile PBS and plated onto blood agar plates. Plates were incubated for 48 h and colonies were counted to obtain the bacterial viability measured in CFU/mL. All tests were performed in triplicates and in three independent runs (i.e. n = 3 × 3 or n = 9).

### Confocal laser scanning microscopy (CLSM)

A second set of RB disks (0, 0.05, 0.10 wt% curcumin) were produced as previously reported. Biofilms were formed for 6 h or 24 h, treated (“Light”) or not (“Dark”) with light, stained with live/dead bacterial viability kit (Baclight Bacterial Viability kit L7012; Thermo Fisher Scientific, Waltham, MA, USA) and incubated in dark at room temperature for 15 min to allow penetration of the fluorophores inside the bacterial cells. Specimens were washed twice with 1× PBS and examined under a Leica SP8 Resonant-scanning confocal/multiphoton microscope using Leica Fluotar VISIR 25×/0.95 water objective, with free working distance of 2.3 mm.

### Curcumin release from resin disks

Additional resin disks (6 mm-diameter × 1 mm-thickness) for all optimal formulations were prepared as previously reported using PVS molds and the Valo LCU (830 mW/cm^2^). To match biofilm formation conditions, disks were added to a 24-well plate and incubated in 0.5 mL 37 °C MQ ultrapure-water for 6 h or 24 h. After the designated duration in water, the disks were removed and the extract processed using an acetone-buffer system and a plate reader (BioTek, Winooski, VT, USA) at OD_520nm_^64^. The standard curve was built using a stock solution of 100 ppm curcumin in 100% Acetone and diluting with an acetone-buffer solution (of 10 mL pure acetone: 10 mL 0.05 M NaHCO_3_: 1 mL 0.1 M NaOH) to 0.01–15 ppm. The samples were diluted by a factor of 2 with acetone-buffer prior to recording the OD_520nm_ and calculating the curcumin concentration released (n = 6). Eluent from unloaded RB disks (0% curcumin) served as a test control to correct for any other eluted components with similar OD520nm peak. The detection limit for this assay is ~ 0.01 ppm.

### Data analysis

All statistical analysis was conducted using IBM SPSS Statistics for Windows, Version 28.0 software package (IBM Corp., Armonk, New York, US; source: https://www.ibm.com/products/spss-statistics). The data was analyzed with a multi-factor univariate general linear model and post-hoc Tukey (α = 0.05). Data was presented as the mean ± one standard deviation. Statistical significance was accepted as p < 0.05.

## Data Availability

The authors confirm that the data supporting the findings of this study are available within the article and may be further provided upon reasonable request to Dr. Adriana Manso (amanso@dentistry.ubc.ca)*.*
